# 2022 Update to state of the journal of patient-reported outcomes

**DOI:** 10.1186/s41687-022-00412-7

**Published:** 2022-01-12

**Authors:** Ron D. Hays, Chih-Hung Chang

**Affiliations:** 1 University of California, Los Angeles, USA; 2 Washington University School of Medicine, St. Louis, USA

The *Journal of Patient-Reported Outcomes* (JPRO) is an international, open access, multi-disciplinary journal publishing original research and review articles, brief communications, commentaries, editorials, and reviews of recent books and software advances in the field of patient-reported outcomes. The JPRO is an official journal of the International Society for Quality of Life Research (ISOQOL). The first JPRO articles were published on September 12, 2017.

We want to acknowledge the immense contributions of Dennis Revicki, JPRO Founding Co-Editor-in-Chief, who died on May 9, 2021. His impact on ISOQOL and the field of patient-reported outcomes was immense. We miss him greatly.

The fifth year (2021) of the JPRO was another successful one due to the contributions made by reviewers and the consistently high quality of the science reflected in the published articles. We are also extremely grateful for the conscientious work of the associate editors: Jill Carlton (*University of Sheffield, UK),* Joanne Greenhalgh (*University of Leeds, UK),* John Devin Peipert (*Northwestern University, USA),* Caroline Potter (*University of Oxford, UK), and* Jessica Roydhouse (*University of Tasmania, Australia).*

We are confident in a very positive trajectory for the JPRO in 2022 and we encourage those conducting patient-reported outcomes research to submit manuscripts and to serve as peer reviewers.

Sincerely,

Ron D. Hays and Chih-Hung Chang

JPRO Co-Editors-in-Chief

